# Exploring dynamic change in arterial base excess with patient outcome and lactate clearance in the intensive care unit by hierarchical time-series clustering

**DOI:** 10.3389/fmed.2022.1020806

**Published:** 2022-11-08

**Authors:** Dongkai Li, Shengjun Liu, Jiahui Zhang, Wei Cheng, Jiayu Mao, Na Cui

**Affiliations:** Department of Critical Care Medicine, Peking Union Medical College Hospital, Beijing, China

**Keywords:** hyperlactatemia, base excess, lactate clearance, resuscitation, critical care, mortality

## Abstract

**Background:**

Hyperlactatemia is common in the intensive care unit (ICU) and relevant to prognosis, while the process of lactate normalization requires a relatively long period. We hypothesized that the dynamic change in base excess (BE) would be associated with ICU mortality and lactate clearance.

**Methods:**

We performed a retrospective cohort study of adult patients with hyperlactatemia admitted to the ICU from 2016 to 2021. The patients were divided into two groups according to whether the peak BE in 12 h was reached in the first 6 h. We compared ICU mortality and lactate clearance at 6 and 12 h after ICU admission.

**Results:**

During the study period, 1,608 patients were admitted to the ICU with a lactate concentration of >2.0 mmol/L and stayed in the ICU for >24 h. The mortality rate was 11.2%. The patients were divided into two groups according to whether the peak BE was reached in the first 6 h following ICU admission: Peak BE12h ≤ 6h and Peak BE12h > 6h. The patients were also recorded as whether bicarbonate treatment was received (bicarbonate group, CRRT included) or not (non-bicarbonate group). Furthermore, lactic acid clearance patterns were identified by time-series clustering (TSC) using various algorithms and distance measures. We compared ICU mortality and lactate clearance at 6 and 12 h after ICU admission with logistic regression. After adjustment for other confounding factors, we found that Peak BE12h > 6h was independently associated with ICU mortality with an odds ratio of 2.231 (*p* = 0.036) in the bicarbonate group and 2.359 (*p* < 0.005) in the non-bicarbonate group. In addition, based on the definition of >10% lactate clearance at 6 h or >30% at 12 h, we found that Peak BE12h ≤ 6h had 85.2% sensitivity and 38.1% specificity for effective lactate clearance. In time-series clustering analysis, four categories were discriminated, and pattern of lactic acid clearance reveals the early prognostic value of BE in clearance of lactic acid.

**Conclusion:**

A prolonged time to reaching the peak BE was independently associated with ICU mortality. In patients with hyperlactatemia, Peak BE12h ≤ 6h could be used as an indicator to predict effective lactate clearance.

## Introduction

Hyperlactatemia has been traditionally considered a signal of anaerobic metabolism mainly induced by tissue hypoperfusion and hypoxia (1). Hyperlactatemia is a common post-surgery condition in the intensive care unit (ICU). Both the lactate level and lactate clearance are considered important predictors of post-surgery patient outcomes in critical care settings (2–5) and are therapeutic targets in the process of resuscitation and intervention (6).

As the adverse effects of resuscitation, such as fluid overload and pulmonary edema, have continuously emerged in the critical care setting in recent years (7, 8), intensivists are confronted with the urgency of identifying which patients will not benefit from more resuscitation, especially patients with hyperlactatemia. From a physiological viewpoint, persistent hyperlactatemia may represent a state of disequilibrium between increased production and impaired clearance. Adequate resuscitation should aim to suppress the excess production rather than achieve absolute lactate normalization, the latter of which may take more than 24 h to achieve (9). Conversely, hyperlactatemia, which corresponds to the accumulation of lactate rather than lactic acidosis (10), may not indicate the existence of metabolic acidosis or the efficacy of treatment. The base excess (BE) is based on the quantification of the change in the metabolic acid–base status and can be measured by most modern blood gas machines. The BE is one of the most extensively studied prognostic markers used to evaluate trauma patients in the critical care setting (11) and should be considered an adjunctive indicator in the intervention.

## Materials and methods

The data collection for this study was approved by the Institutional Ethics Committee of Peking Union Medical College Hospital (PUMCH; approval number: JS-1170), which waived the need for informed consent.

**FIGURE 1:**
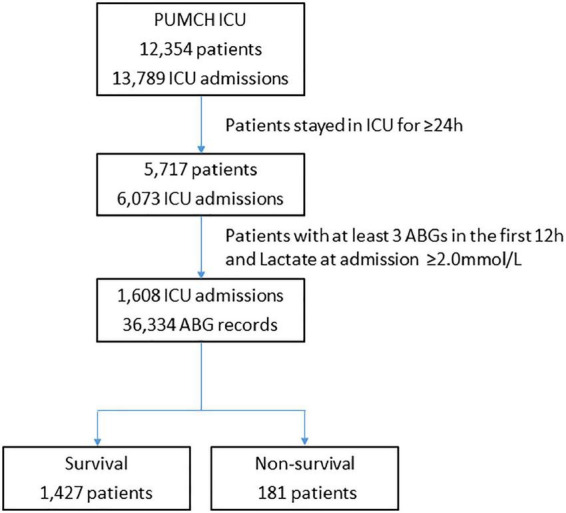
Flowchart showing step-by-step selection of patients enrolled in the study. ICU, intensive care unit; ABG, arterial blood gas.

We retrospectively examined all blood gas data from more than 1,600 consecutive critically ill patients with hyperlactatemia (lactate concentration of ≥2.0 mmol/L in the arterial blood gas at admission) who were admitted to the ICU, underwent at least three arterial blood gas tests during the first 12 h after ICU admission, and stayed for more than 24 h from May 2016 until June 2021 (Figure 1). The data required for analysis were collected from our local clinical care database and were available for computer-based retrieval. We also retrospectively obtained demographic data [age, sex, Acute Physiology and Chronic Health Evaluation (APACHE) II score], treatment data [use of a ventilator, vasoactive agents, bicarbonate sodium, and continuous renal replacement therapy (CRRT)], and outcome data from these records.

Arterial blood samples were collected at the time of ICU admission and during the subsequent clinical course (12 h after ICU admission) when necessary in heparinized blood gas syringes (safePICO sampler; Radiometer, Copenhagen, Denmark) and analyzed in one of three ICU blood gas analyzers [Radiometer ABL90 Flex blood gas analyzer (Radiometer) or the GEM^®^ Premier™ 3,000 or 4,000 analyzer (Instrumentation Laboratory, Lexington, MA, USA)]. All analyzers underwent routine internal quality control before performing the tests. Each analyzer measured samples at 37°C. Nursing staff from the ICU who had been trained to use the machine by support staff performed the blood analysis. Samples were processed within 1 h of being drawn. All data were stored in computerized records. We collected data regarding the pH, lactate, and BE from the analyzer output.

The sampling time of the peak recorded arterial BE (the minimum value of BE) in the first 12 h following admission to the ICU was considered the time of Peak BE12h. The patients were then divided into two groups according to whether the Peak BE12h occurred in the first 6 h following admission to the ICU: Peak BE12h ≤ 6h or Peak BE12h > 6h. Considering the significant impact on treatment of acid–base disturbances from bicarbonate sodium infusion and CRRT, which are the fundamental and indispensable treatments in critical care settings, the patients were recorded as whether bicarbonate treatment was received (bicarbonate group, CRRT included) or not (non-bicarbonate group). No Ringer’s lactate solution was infused during treatment.

Clustering is a data mining technique where similar data are placed into related or homogeneous groups without advanced knowledge of the groups’ definitions. Time-series clustering is a special type of clustering. The concept of time-series clustering is given a dataset of n time-series data D = {F1, F2, … Fn}, the process of unsupervised partitioning of D into C = {C1, C2, … Ck}, in such a way that homogenous time series are grouped together based on a certain similarity measure. In this study, hierarchical clustering of time series was used to make clustering analysis. Hierarchal clustering (12) is an approach of cluster analysis which makes a hierarchy of clusters using agglomerative or divisive algorithms. In this research, agglomerative hierarchical clustering was used. Four steps are applied: (1) start assigning each observation as a single point cluster; (2) find the closest (most similar) pair of clusters and make them into one cluster; (3) find the two closest clusters and make them to one cluster; (4) repeat steps 2 and 3 until all observations are clustered into one single cluster of size N. Euclidean distance was used to identify similar and dissimilar measures. Dynamic time warping (DTW) is used to detect and match the internal patterns of two time series of the same size by calculating a two-dimensional distance matrix with all possible pairwise Euclidean distances between time points. DTW alignment is determined by finding the shortest path that minimizes the overall combined values of the distance matrix.

### Outcome

The primary outcome was ICU mortality, which included death in the ICU, do-not-resuscitate intention, and discharge without medical advice. The secondary outcome was the lactate clearance at 6 and 12 h following ICU admission, which was calculated as follows: [(initial lactate − second lactate (at 6 or 12 h))/initial lactate] × 100%. The standard of effective lactate clearance was defined as ≥10% lactate clearance at 6 h or ≥30% clearance at 12 h. The duration of ICU stay was also recorded.

### Statistical analysis

The statistical package SPSS 22.0 for Windows (IBM Corp., Armonk, NY, USA) was used for statistical analyses. Data are presented as mean ± standard deviation or median (interquartile range) as indicated by assessment of a normal distribution (D’Agostino–Pearson omnibus normality test). In univariate analyses, the Mann–Whitney *U*-test was used to compare continuous variables, while Fisher’s exact test was used to compare categorical variables between patients who did and did not survive. All tests were two-sided, and *p* < 0.05 indicated statistical significance. Logistic regression was performed to evaluate the association of Peak BE12h > 6h with ICU mortality and effective lactate clearance, with adjustment for the APACHE II score, lactate concentration, and BE at admission.

## Results

### Patient characteristics

During the study period, 1,608 patients were admitted to the ICU with a lactate concentration of >2.0 mmol/L and stayed in the ICU for ≥24 h (Figure 1). During the first 12 h, the illness of the patients in the non-survival group was more severe than that in the survival group [APACHE II score: 27 (21–35) vs. 16 (12–21), *p* < 0.001], and the non-surviving patients underwent more arterial blood gas tests (6.3 ± 2.2 vs. 5.3 ± 1.8, *p* < 0.001) and ICU treatments, as shown in Table 1. Although more patients in the non-survival group underwent treatment with CRRT and bicarbonate sodium, a lower percentage of surviving patients had Peak BE12h ≤ 6h.

**TABLE 1:** General characteristics and clinical outcomes in the survival and non-survival groups.

Characteristics	Survival (*n* = 1,427)	Non-survival (*n* = 181)	*P*-value
**Demographic**			
Age, years	54.6 ± 17.2	58.1 ± 16.2	0.007
Gender, male (%)	744 (52.1%)	99 (54.7%)	0.516
Admission status			
Elective surgery (%)	983 (68.9%)	100 (55.2%)	<0.001
Emergency surgery (%)	247 (17.3%)	22 (12.2%)	<0.001
Medical emergency (%)	159 (11.1%)	58 (32.0%)	<0.001
APACHEII [IQR]	16 [12, 21]	27 [21, 35]	<0.001
ABG counts during the first 12 h	5.3 ± 1.8	6.3 ± 2.2	<0.001
**ABG at admission**			
pH	7.372 ± 0.089	7.359 ± 0.117	0.152
Lac, mmol/L	4.7 ± 3.1	6.0 ± 5.2	<0.001
BE, mmol/L	−3.2 ± 4.6	−4.9 ± 6.9	<0.001
**Treatment received**			
Mechanical ventilation (%)	1,140 (79.9%)	147 (81.2%)	0.674
Vasoactive agents (%)	923 (64.7%)	155 (85.6%)	<0.001
CRRT (%)	51 (3.6%)	31 (17.1%)	<0.001
Bicarbonate sodium infusion (%)	399 (28.0%)	93 (51.4%)	<0.001
**Hemodynamic**			
Heart rate, bpm	97.4 ± 17.6	105.4 ± 20.2	<0.001
Mean arterial pressure, mmHg	90.8 ± 10.9	87.0 ± 11.8	<0.001
Fluid balance in the first 12 h, mL [IQR]	579 [−125, 1374.5]	841 [−139.5, 2126]	<0.001
**Outcome**			
Lactate clearance at 6 h,% [IQR]	16.1% [−16.9%, 43.2%]	10.8% [−23.7%, 28.9%]	0.003
Lactate clearance at 12 h,% [IQR]	40.0% [11.9%, 61.7%]	24.8% [−24.1%, 47.0%]	<0.001
**Peak Value during the first 12 h**			
Lactate, mmol/L	5.5 ± 3.5	8.0 ± 6.8	<0.001
BE, mmol/L	−5.2 ± 4.6	−7.5 ± 7.1	<0.001
Peak BE12h ≤ 6h (%)	1,155 (80.9%)	124 (68.5%)	<0.001
ICU duration, hours	71 [42, 143]	140 [55, 273]	<0.001

Results are presented as mean (standard deviation), median [IQR], or *n* (%). Lac, lactate; BE, base excess; APACHE II, Acute Physiology and Chronic Health Evaluation II; IQR, interquartile range; Peak BE12h ≤ 6h, the peak value (minimal) BE value during the first 12 h after admission occurred in the first 6 h; ABG, arterial blood gas; CRRT, continuous renal replacement therapy; ICU, intensive care unit.

### Multivariable analysis on ICU mortality

The general ICU mortality rate among the enrolled patients was 11.2% (*n* = 181). Logistic regression showed that the APACHE II score and Peak BE12h > 6h were independently associated with ICU mortality with an odds ratio of 1.136 (*p* < 0.005) and 2.231 (*p* = 0.036) in the bicarbonate group and 1.168 (*p* < 0.005) and 2.359 (*p* < 0.005) in the non-bicarbonate group. As shown in Table 2, neither lactate nor BE at ICU admission were significantly associated with ICU survival, nor were the peak values of lactate and BE.

**TABLE 2:** Association between peak BE12h > 6h and ICU mortality.

Parameters	Unadjusted OR	Adjusted OR	(95% CI)	*p*
**Group without bicarbonate sodium used**				
Age	1.012	0.994	(0.980, 1.008)	0.387
Gender	0.802	0.821	(0.506, 1.333)	0.426
Apache II	1.164	1.168	(1.130, 1.208)	< 0.005
Peak BE12h > 6 h	2.102	2.359	(1.306, 4.260)	< 0.005
Lactate at admission	0.968	0.838	(0.618, 1.135)	0.253
BE at admission	1.044	0.911	(0.791, 1.051)	0.201
Peak lactate in first 6 h	0.996	1.140	(0.845, 1.538)	0.391
Peak BE in first 6 h	1.042	1.118	(0.964, 1.295)	0.140
**Group with bicarbonate sodium used**				
Age	1.018	1.010	(0.993, 1.027)	0.247
Gender	1.147	1.561	(0.880, 2.768)	0.561
Apache II	1.146	1.136	(1.099, 1.174)	< 0.005
Peak BE12h > 6h	1.957	2.231	(1.054, 4.725)	0.036
Lactate at admission	1.059	0.975	(0.874, 1.088)	0.653
BE at admission	0.939	0.976	(0.894, 1.064)	0.576
Peak lactate in first 6 h	1.096	1.051	(0.950, 1.162)	0.335
Peak BE in first 6 h	0.878	0.968	(0.868, 1.079)	0.555

OR, odds ratio; CI, confidence interval; ICU, intensive care unit; APACHE II, Acute Physiology and Chronic Health Evaluation II; Peak BE12h > 6h, the peak value (minimal) BE value during the first 12 h after admission occurred after the first 6 h; BE, base excess.

### Association of peak BE12h ≤ 6h with lactate clearance

**FIGURE 2:**
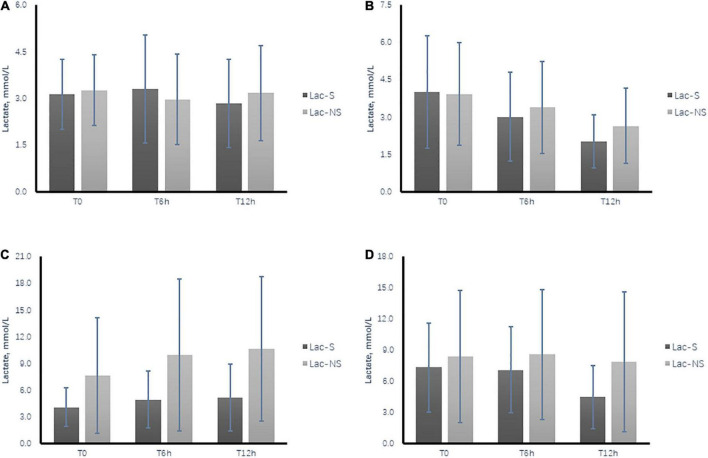
Time course of dynamic changes in arterial lactate level during the first 12 h after ICU admission between the survival group (Lac-S) and non-survival group (Lac-NS). **(A)** Subgroup of patients without bicarbonate infusion and Peak BE12h > 6h. **(B)** Subgroup of patients without bicarbonate infusion and Peak BE12h ≤ 6h. **(C)** Subgroup of patients with bicarbonate infusion and Peak BE12h > 6 h. **(D)** Subgroup of patients without bicarbonate infusion and Peak BE12h ≤ 6h. T0, at admission; T6h, 6 h after ICU admission; T12h, 12 h after ICU admission.

In the survival group, the lactate level at admission was 4.7 ± 3.1 mmol/L, with a 6-h lactate clearance of 16.1% (−16.9% to 43.2%) and 12-h clearance of 40.0% (11.9%–61.7%). These results were superior to those in the non-survival group, with lactate of 6.0 ± 5.2 mmol/L at admission, 6-h clearance of 10.8% (-23.7% to 28.9%), and 12-h clearance of 24.8% (-24.1% to 47.0%) (Table 1 and Figure 2).

Based on the definition of effective lactate clearance (≥10% at 6 h or ≥30% at 12 h), the results showed that Peak BE12h ≤ 6h had 85.2% sensitivity and 38.1% specificity for effective lactate clearance (Supplementary Table 1). Comparison of the lactate time course between the subgroups confirmed that the lactate levels were lower in the survival than non-survival group. Meanwhile, in subgroups with Peak BE12h ≤ 6h, an obvious tendency toward lactate clearance was shown in both the survival and non-survival groups, while the opposite tendency was shown in the subgroups with Peak BE12h > 6h (Figure 2).

### Sensitivity analysis

**FIGURE 3:**
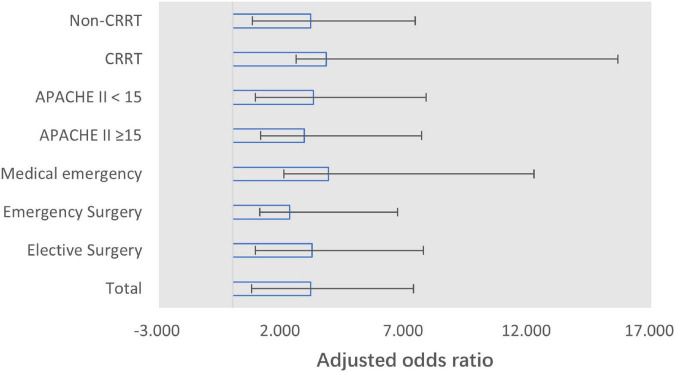
Forest plot for adjusted odds ratio of Peak BE12h and effective lactate clearance (6-h lactate clearance of ≥10% or 12-h lactate clearance of ≥30%) per subgroup. Adjusted for APACHE II score, lactate, and base excess at admission. CRRT, continuous renal replacement therapy.

In the sensitivity analysis, to examine whether the association between Peak BE12h ≤ 6h and effective lactate clearance remained across different subsets, we explored the association among groups of patients with or without CRRT treatment, APACHE II at ICU admission below or above 15 points, and admission type with medical emergency, emergency surgery, or elective surgery. The results showed that, compared with the general patients, the association remained among various of subgroups, while the odds ratio tends to increase in patients with greater illness severity [APACHE II score of ≥15 subgroup: 2.907 (1.773–4.767); CRRT subgroup: 3.807 (1.226–11.825); medical emergency subgroup: 3.893 (1.815–8.351)] (Figure 3).

### Time-series clustering analysis

**FIGURE 4:**
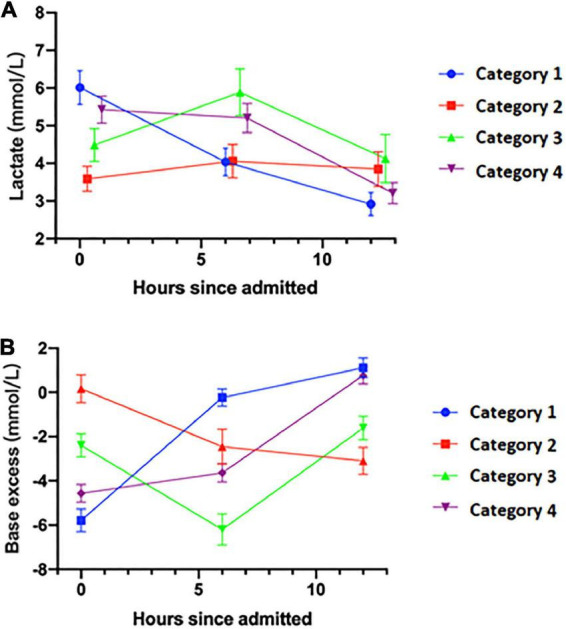
Lactate and base excess level of four categories of patients in the first 12 h. **(A)** Dynamic change of lactate level. **(B)** Dynamic change of base excess level. *X*-axis, hours since admitted, hours.

As is shown in Figure 4A, four categories of lactate clearance patterns were found, of which the clinical characteristics are shown in Supplementary Table 2. In Category 1 and Category 4, lactic acid decreases as the time goes on. However, compared to Category 1, patients belonging to Category 4 have a higher lactate level and lower lactate clearance rate. In Category 2 and Category 3, lactate increase in the first 6 h. However, compared to Category 2, patients belonging to Category 3 have a greater lactate level in 6 h after admission and decrease more rapidly in 12 h. After revealing the base excess (BE) level of four groups (Figure 4B), BE in Category 1 and Category 4 increases as the time goes, which indicates relief of metabolic acidosis. Consequently, lactate level of these two groups did not abnormally elevate during the resuscitation process. Besides, the analysis on variation tendency also indicated the individual feature of the dynamic change of lactate and BE level among the four categories, as shown in Supplementary Figure 1.

## Discussion

This study investigated the association between the time to reaching the peak BE (indicated by Peak BE12h ≤ 6h and Peak BE12h > 6h) during the first 12 h after ICU admission and the outcomes of patients. Our results showed that a prolonged time to the peak BE was independently associated with increased mortality in the ICU. Meanwhile, Peak BE12h ≤ 6h behaved as an effective predictor of effective lactate clearance at 6 or 12 h after ICU admission.

Hyperlactatemia has been traditionally considered a signal of tissue hypoperfusion; consequently, the presence of hyperlactatemia will urge the intensivist to implement continuous volume resuscitation and various other treatments until the lactate concentration decreases to a normal level (7, 13, 14). Perfusion-related indicators exhibit markedly different dynamic change rates in the clinical process. A study of survivors of septic shock showed that compared with other perfusion-related variables, such as the central venous oxygen saturation, central venous–arterial pCO2 gradient, and capillary refill time, the lactate normalization rate decreased significantly after the first 6 h and only half of the patients achieved a normal lactate level at 24 h (7). Lactate clearance was then studied to evaluate the dynamic change in the lactate dynamic concentration within a specific period (6 or 12 h), and lactate clearance was shown to be associated with patient outcomes in the ICU (3). Nevertheless, lactate clearance still requires a relatively long period of time for evaluation, and the optimal cutoff value is controversial (2, 7). As the requirements for early goal-directed therapy in patients with sepsis (4) and fluid management strategies (9) in the ICU become increasingly urgent, a novel indicator with higher accessibility and sensitivity is required for resuscitation monitoring and avoidance of over-resuscitation in critical settings.

Base excess is the amount of base in millimoles required to titrate one of whole arterial blood to a pH of 7.40 with sample fully saturated with oxygen at 37°C and a PaCO2 of 40 mmHg. In critically ill patients, a negative BE or increasingly negative BE was shown to be predictive of volume resuscitation and shock-related complications, including a prolonged ICU stay, renal injury, acute lung injury, and multiorgan failure (15–18). The blood lactate level and standard BE are commonly used circulating markers of the tissue hypoperfusion and latent systemic metabolic acidosis (19, 20). In fact, hyperlactatemia may occur with or without concurrent metabolic acidosis because when hyperlactatemia occurs without worsening tissue perfusion, the buffering mechanisms may compensate for any fall in pH. Considering the controversial opinion on the bicarbonate therapy on severe metabolic acidosis (21), the clinical significance of dynamic change of negative BE, which is more common in the critical care settings than severe acidosis, as well as its response to the treatment including the bicarbonate or CRRT therapy, was uninvestigated.

In this study, we showed that the dynamic change in BE was associated with that in lactate, and an earlier time to the peak BE (Peak BE12h ≤ 6h) during the first 12 h after ICU admission is predictive of effective lactate clearance and better outcomes in critical care settings. The multivariable analysis showed that Peak BE12h > 6h and <10% lactate clearance at 6 h after ICU admission were independently associated with higher mortality in the ICU. Our results confirm the significance of the evaluation of the dynamic change in BE and are consistent with previous studies (18, 21, 22). Notably, this association did not change in the bicarbonate group, which involved intervention to achieve acid–base equilibrium with bicarbonate sodium or CRRT. The temporary change in the acid–base equilibrium, instead of essential hypoperfusion, might explain this finding, and neither the dynamic change in the BE nor the outcome could be reversed by bicarbonate or CRRT. This is consistent with the findings of a recent study on the application of bicarbonate therapy in patients with metabolic acidemia (21), which advocated that the active correction on metabolic acidosis may not be associated with better clinical outcome.

Our results also showed that Peak BE12h ≤ 6h was clinically useful in predicting effective lactate clearance, or lactate normalization, in critically ill patients with a sensitivity of 85.2% in the bicarbonate group and 87.9% in the non-bicarbonate group, as shown in Supplementary Table 1. As far as we know, this study is the first to evaluate the association between the dynamic change in BE and the process of lactate normalization. As a retrospective study, our results cannot further interpret the potential benefits of dynamic change in BE or compared the two resuscitation strategies based on different targets, which require prospective controlled study to confirm. Nevertheless, considering the great advantage of the Peak BE12h in the early identification of patients who had benefitted from the previous resuscitation process than lactate normalization or clearance, avoidance of unnecessary therapy involving fluids or vasoactive/inotrope drugs may significantly improve the ICU outcome of these patients and relieve the workload of the ICU team.

Our sensitivity analysis of different subgroups also supported this conclusion. The patients with more severe illness (subgroup with APACHE II score of ≥15, CRRT, and medical emergency) showed a greater association between the Peak BE12h ≤ 6h and effective lactate clearance. This could be attributed to the higher baseline lactate level (at admission) and the immediate response to the treatment. The time course of lactate and BE during the first 12 h also supports the conclusion that Peak BE12h ≤ 6h is of value in the prediction of dynamic changes in lactate within 6 and 12 h, regardless of bicarbonate application or CRRT.

Our study has some limitations. First, this study was limited by its retrospective nature and patients’ characteristics. Thus, no causal relationship could be established. Meanwhile, although the relatively large dataset balanced the individualized treatment factors among different patients, confounding factors other than the use of bicarbonate, such as the comorbidity of chronic kidney insufficiency or chronic obstructive pulmonary disease, may have influenced the validity of Peak BE12h ≤ 6h. Second, as shown in the results in bicarbonate group, deliberately altering the acid–base equilibrium will not change the ICU outcome or lactate clearance. As a result, Peak BE12h ≤ 6h should not be used as a “therapeutic target” but instead as a signal indicator in treatment evaluation during the first 6 h. Third, in our study, the definition of effective lactate clearance was established based on comprehensive consideration of previous studies (2, 3). Different viewpoints on the cutoff value of effective lactate clearance still exist, and our definition might be inappropriate in some specific subgroups of patients and clinical settings.

In time-series clustering, hierarchical clustering is a well-validated clustering method. Instead of K-means or SVM, we do not have to make assumptions of number of groups before the analysis when applying hierarchical clustering. Also, compared to traditionally used Euclidean distance, dynamic time warping distance considers different treatments of each patient over time. The biggest advantage of dynamic time warping is that it allows time scaling during matching, so it can better find the best matching sequence in a bunch of sequence sets.

## Conclusion

In conclusion, this study showed that a prolonged time to reach the peak BE is associated with worse outcomes in ICU. In patients with hyperlactatemia, Peak BE12h ≤ 6h has the potential to be an adjunctive indicator to predict effective lactate clearance at 6 and 12 h after ICU admission. Whether patients received bicarbonate treatment did not influence the association of dynamic changes in BE with patient outcomes and effective lactate clearance.

## Data availability statement

The raw data supporting the conclusions of this article will be made available by the authors, without undue reservation.

## Ethics statement

The studies involving human participants were reviewed and approved by Institutional Ethics Committee of Peking Union Medical College Hospital. The patients/participants provided their written informed consent to participate in this study.

## Author contributions

DL and NC: conceptualization. DL and SL: validation and writing—original draft preparation. WC: investigation. DL and JM: data curation. NC: writing—review and editing. All authors read and agreed to the published version of the manuscript.
