# Supplementary material for: Exploring dynamic change in arterial base excess with patient outcome and lactate clearance in the intensive care unit by hierarchical time-series clustering
Source: Front Med (Lausanne). 2022 Nov 8;9:1020806. doi: 10.3389/fmed.2022.1020806 (PMC9679290; doi:10.3389/fmed.2022.1020806)
Supplement: Supplementary file 1 [file Data_Sheet_1.PDF]

Supplement Figure S1. Lactate ad base excess variation tendency of four categories. Yellow shaded area: Lactate level; Blue shaded area: base excess level. Panel (a)-(d) refers to the Category A to D.

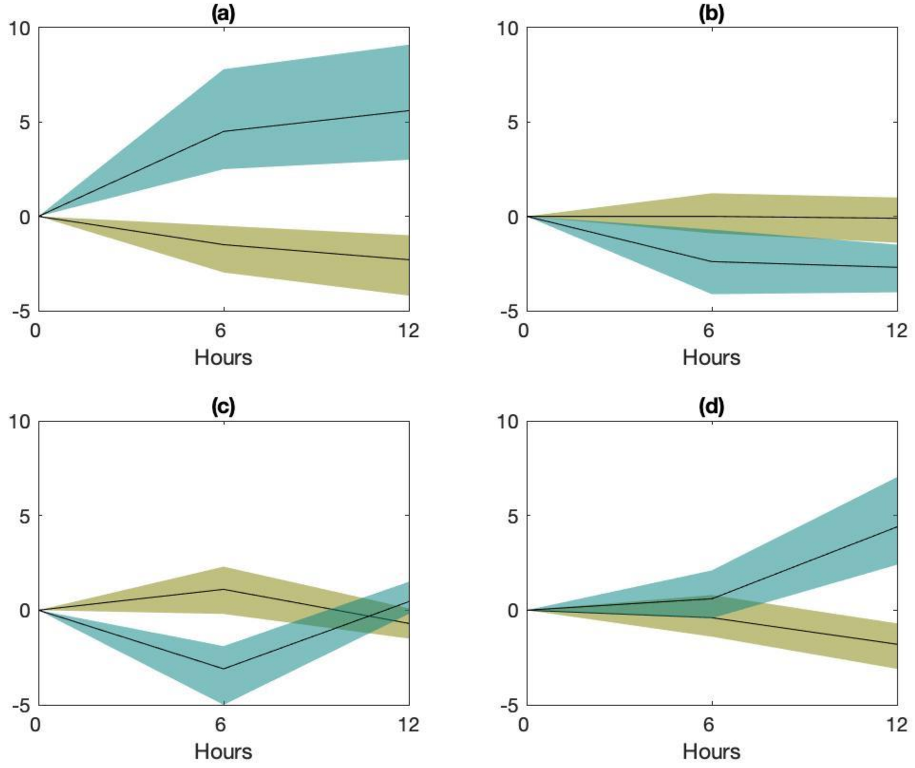

Table S1. Predictive performance of Peak BE_{12h}≤6h for effective lactate clearance in critically ill patients

		Effective lactate clearance (6h ≥10% or 12h≥30%)		Sensitivity (%)	Specificity (%)	PPV (%)	NPV (%)
		No	Yes				
Bicarbonate Group	Peak BE_{12h}≤6h	182	697	85.2（82.5, 87.5）	38.1（32.6, 43.9）	79.3 (76.4, 81.9)	48.1 (41.5, 54.7)
	Peak BE_{12h}>6h	112	121				
Non-bicarbonate Group	Peak BE_{12h}≤6h	148	248	87.9% (83.4, 91.4)	29.5 (23.5, 36.3)	62.6（57.6, 67.4）	64.6 (54.1, 73.9)
	Peak BE_{12h}>6h	62	34				

PPV, positive predictive value; NPV, negative predictive value

Table S2. Clinical characteristics of each category in in critically ill patients

	Category 1	Category 2	Category 3	Category 4
**Female (%)**	164 (47.3)	116 (54.5)	83 (48.8)	172 (44.7)
**Lac T0h**	4.7(3.1,7.3)	2.9(2.3,4.05)	3.55(2.9,5)	4.2(3.55,6.95)
**Lac T6h**	2.8(1.7,4.9)	3.3(2.1,4.85)	4.7(3.3,7.05)	3.9(4.7,7.4)
**Lac T12h**	2(1.2,3.4)	3(1.8,4.6)	2.7(3,4.625)	2.3(2.7,3.8)
**BE T0h**	-5.4(-8.8,-2.5)	-0.1(-2.6,1.9)	-2.4(-0.1,-0.6)	-4.4(-2.4,-1.8)
**BE T6h**	-0.5(-2.6,2.1)	-2.7(-5.6,0.4)	-5.7(-2.7,-3.7)	-3.6(-5.7,-1.1)
**BE T12h**	1.2(-1.5,3.4)	-2.9(-5.7,-0.6)	-1.7(-2.9,0.3)	0.5(-1.7,3)
**pH T0h**	7.33(7.28,7.39)	7.44(7.38,7.49)	7.39(7.44,7.44)	7.35(7.39,7.41)
**pH T6h**	7.41(7.37,7.45)	7.39(7.35,7.45)	7.38(7.34,7.39)	7.36(7.34,7.41)
**pH T12h**	7.43(7.39,7.46)	7.38(7.33,7.43)	7.39(7.38,7.42)	7.42(7.39,7.45)
**APACHE-II**	18(13,23)	18(14,23)	17(18,24)	17(17,24)
**Age**	55(41,65)	61(52,70.5)	60.5(61,70)	56(60.5,67)
